# IRAK1 is a novel DEK transcriptional target and is essential for head and neck cancer cell survival

**DOI:** 10.18632/oncotarget.6028

**Published:** 2015-10-26

**Authors:** Allie K. Adams, Lyndsey C. Bolanos, Phillip J. Dexheimer, Rebekah A. Karns, Bruce J. Aronow, Kakajan Komurov, Anil G. Jegga, Keith A. Casper, Yash J. Patil, Keith M. Wilson, Daniel T. Starczynowski, Susanne I. Wells

**Affiliations:** ^1^ Division of Oncology, Cincinnati Children's Hospital Medical Center, Cincinnati, OH, USA; ^2^ Division of Experimental Hematology and Cancer Biology, Cincinnati Children's Hospital Medical Center, Cincinnati, OH, USA; ^3^ Division of Biomedical Informatics, Cincinnati Children's Hospital Medical Center, Cincinnati, OH, USA; ^4^ Department of Otolaryngology, Head and Neck Surgery, University of Cincinnati, Cincinnati, OH, USA; ^5^ Department of Cancer Biology, University of Cincinnati, Cincinnati, OH, USA

**Keywords:** DEK, IRAK1, HNSCC, HPV, RNA-Seq

## Abstract

The chromatin-binding DEK protein was recently reported to promote the growth of HPV^+^ and HPV^−^ head and neck squamous cell carcinomas (HNSCCs). Relevant cellular and molecular mechanism(s) controlled by DEK in HNSCC remain poorly understood. While DEK is known to regulate specific transcriptional targets, global DEK-dependent gene networks in HNSCC are unknown. To identify DEK transcriptional signatures we performed RNA-Sequencing (RNA-Seq) in HNSCC cell lines that were either proficient or deficient for DEK. Bioinformatic analyses and subsequent validation revealed that IRAK1, a regulator of inflammatory signaling, and IRAK1-dependent regulatory networks were significantly repressed upon DEK knockdown in HNSCC. According to TCGA data, 14% of HNSCC specimens overexpressed IRAK1, thus supporting possible oncogenic functions. Furthermore, genetic or pharmacologic inhibition of IRAK1 in HNSCC cell lines was sufficient to attenuate downstream signaling such as ERK1/2 and to induce HNSCC cell death by apoptosis. Finally, targeting DEK and IRAK1 simultaneously enhanced cell death as compared to targeting either alone. Our findings reveal that IRAK1 promotes cell survival and is an attractive therapeutic target in HNSCC cells. Thus, we propose a model wherein IRAK1 stimulates tumor signaling and phenotypes both independently and in conjunction with DEK.

## INTRODUCTION

Head and neck squamous cell carcinoma (HNSCC) is a disease comprised of two distinct entities: human papillomavirus (HPV) positive and HPV negative. HPV^−^ disease is attributable to tobacco and alcohol use, and its declining incidence in the US has been ascribed to the well-publicized health risks of these activities. In stark contrast, HPV^+^ disease is on the rise, particularly in younger patient populations [1]. While improved response to traditional chemotherapies and thus favorable long-term survival is observed in HPV+ patients, prognoses remain grim for patients with advanced and metastatic tumors [2]. Furthermore, major quality of life issues arise due to treatment-related tissue damage [3]. Therefore, the need for novel therapeutic targets and biomarkers for both HNSCC subsets must not be underestimated.

DEK is important in various cancer cell types, including breast and bladder cancer, melanoma, and most recently, HNSCCs [4–8]. This is a versatile nuclear protein, with functions that range from chromatin modifier and histone chaperone to modulator of DNA repair, replication, and transcription [9–12]. For example, DEK represses transcription in leukemia cells through inhibition of p300 and P/CAF [13]. DEK also activates transcription via interaction with AP-2α in glioblastoma [14]. Although DEK has been published as a co-activator or co-repressor of transcription in various systems, transcriptome data to determine the role of DEK in global transcriptional regulation in solid tumors is scarce [14–16].

Our previous work highlighted the oncogenic functions of DEK in both HPV^+^ and HPV^−^ human HNSCCs, wherein DEK was highly overexpressed and required for optimal growth and proliferation [8]. Dek loss of function in mice attenuated the proliferation of HPV16 E7 expressing, but not normal, epidermis and inhibited overt tumor growth in a chemically induced model of HNSCC. Furthermore, this work implicated ΔNp63 as a downstream DEK target that regulated DEK-dependent proliferation. In view of the observed specificity of DEK targeting for pre- and overt malignancies, this molecule has been reported as a potential therapeutic target. However, DEK-dependent signaling pathways and molecular mediators of DEK-dependent tumor phenotypes in HNSCC are limited. Herein, we aimed to uncover relevant pathways important in DEK-dependent HNSCC phenotypes that may also be novel therapeutic strategies.

In this study, we performed transcriptome profiling to identify DEK-dependent gene regulatory networks essential for HNSCC. We focused on both subsets of HNSCC, HPV^−^ and HPV^+^, to identify targets that may be beneficial to patients regardless of HPV status. Following gene ontological analysis, biological processes involved in the immune response were strongly implicated. DEK has previously been published as an autoantigen in autoimmune diseases and it can function as a pro-inflammatory protein, suggesting it may regulate inflammatory signaling [17, 18]. Central to the immune response pathway and a significantly repressed target following DEK knockdown is IRAK1, a serine/threonine kinase, which mediates signaling from the toll-like receptor (TLR) and interleukin-1 receptors (IL1R) [19]. The IRAK1 signaling cascade includes the E3 ubiquitin ligase TRAF6, which engages, among other pathways, NF-κB and MAPK signaling. IRAK1 was recently implicated as a novel therapeutic target in myelodysplastic syndrome (MDS) and acute myeloid leukemia (AML), but its function in most solid tumors remains unknown [20]. We found that IRAK1 is overexpressed by genomic amplification and transcriptional up-regulation in a significant proportion of HNSCC tumors. Furthermore, genetic or pharmacologic inhibition of IRAK1 attenuated downstream signaling through TRAF6 and increased apoptosis, suggesting IRAK1 inhibition may be a new therapeutic target in HNSCC. Finally, DEK and IRAK1 contributed to HNSCC survival independently, and targeting them jointly enhanced HNSCC cell death over the targeting of either. Taken together, these data reveal IRAK1 as a component of the DEK transcriptome, and a druggable effector in HNSCC.

## RESULTS

### Profiling the DEK-dependent transcriptome in HNSCC

**Figure 1:**
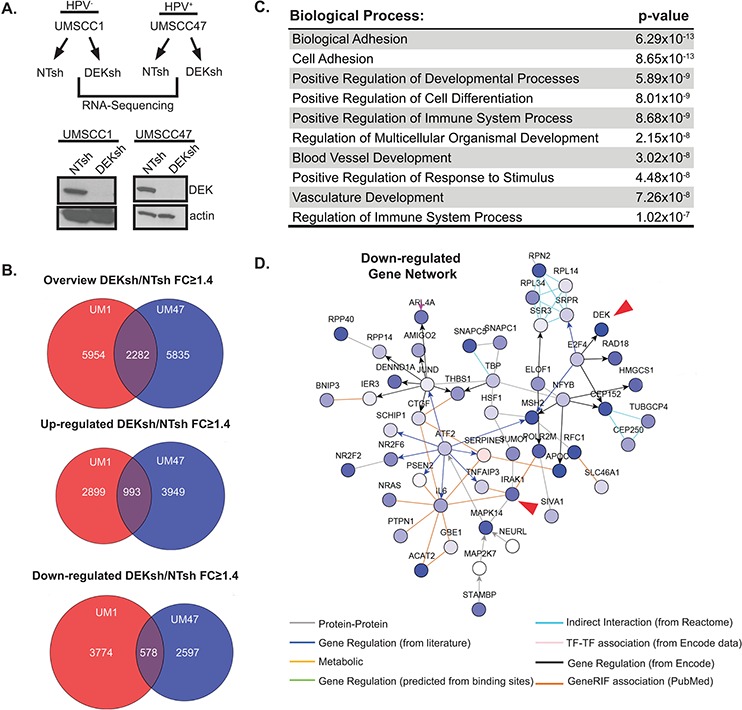
Profiling the DEK-dependent transcriptome in HNSCC **A.** UMSCC1 and UMSCC47 were lentivirally depleted for DEK, as confirmed by western blot analysis, and mRNA from these lines was submitted for RNA-Sequencing (RNA-Seq). **B.** GeneSpring NGS analysis was performed on genes differentially expressed 1.4 fold or greater (DEKsh/NTsh). GeneSpring derived Venn diagrams depict genes altered in both cell lines and overlap highlights genes common to both UMSCC1 and UMSCC47. **C.** Overlapping genes (from panel B, 2282) from UMSCC1 and UMSCC47 were analyzed using ToppGene server to identify significantly altered biological processes. The top 10 biological processes are represented. **D.** A portion of the down-regulated gene networks analyzed on NetWalker highlights nodes containing IRAK1 and DEK in UMSCC1 cells. A full view of view of this map can be found in Supplementary Figure 2.

Little is known about the global impact of DEK loss on gene expression, and relevant transcriptional targets are largely uncharacterized [16]. DEK plays dual roles in transcription, either as a co-activator or a co-repressor depending on the cellular context, and was recently published to bind transcriptional start sites of some of the activated or repressed target genes [12]. In order to define the consequences of DEK loss and identify DEK-dependent transcriptional networks in HNSCC cells, we used a well-established lentiviral approach that was previously published in this model system [8]. The HPV^−^ and HPV^+^ cell lines, UMSCC1 and UMSCC47, respectively, were transduced with DEK versus control knockdown vector and selected with puromycin. Successful knockdown was confirmed by western blot analysis (Figure 1A). Messenger RNA (mRNA) was collected and subjected to RNA-Seq. Independent analyses were performed on UMSCC1 and UMSCC47 RNA-Seq data to identify fold changes for differentially expressed genes. Venn diagrams highlight over 2,000 common genes that were differentially expressed upon DEK loss in these cell lines (Figure 1B). Ontology analyses revealed dysregulation of the immune response pathway (Figure 1C). Specifically, immune response genes were down-regulated in DEK-deficient cells. In parallel, we assessed common transcription factor binding sites among the overlapping gene set, and identified many new sites, along with published ones such as p53 consensus sequences (Supplementary Tables 1–3) [21]. To further define DEK targets relevant in HNSCC, we performed a walk-based network analysis to assign functional interactions between DEK and its transcriptional targets. A portion of the down-regulated gene network for UMSCC1 cells is shown (Figure 1D), with arrows highlighting two interconnected nodes. Expanded versions of this network map, along with others, are found in Supplementary Figures 1–6. DEK down-regulation was connected to many genes of interest, including TNFAIP3, IL6, and MAPKs. Because these genes are well established for their downstream contributions to inflammation and immune signaling, we focused on the most important upstream transducer of toll-like receptor (TLR) and interleukin-1 receptor (IL-1R) signaling that was repressed following DEK loss, IRAK1. Interestingly, a recent publication emphasized the importance of IL1R-dependent signaling in HNSCCs. Therein, signaling from the IRAK1 adapter protein MyD88 was increased in response to erlotinib treatment in EGFR-amplified HNSCCs [22]. Furthermore, inhibition of IL1R signaling enhanced sensitivity to erlotinib treatment, thus supporting clinical potential for the targeting of TLR/IL1R signaling pathways in these cancers.

### DEK regulates IRAK1 mRNA and protein levels

**Figure 2:**
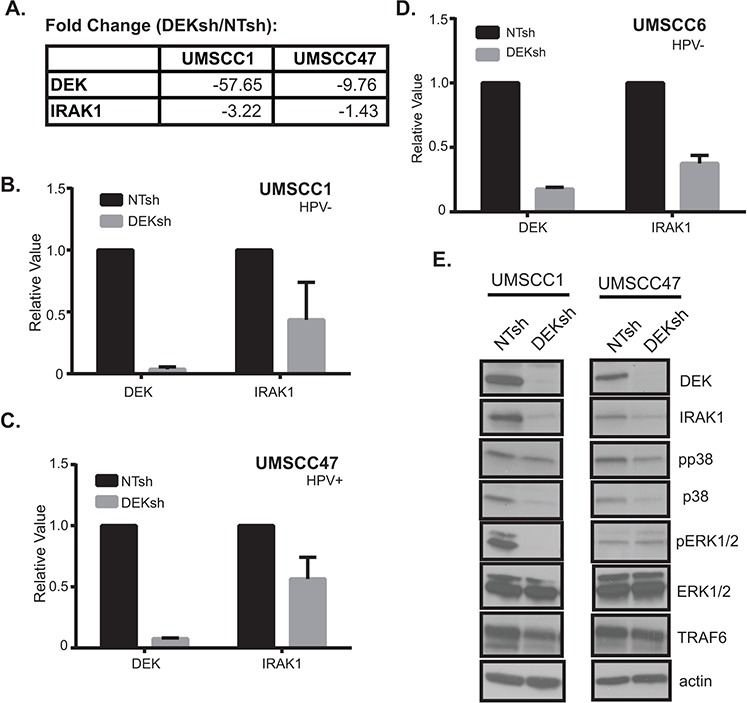
DEK regulates IRAK1 mRNA and protein levels **A.** Table depicts fold changes for DEK and IRAK1 in UMSCC1 and UMSCC47 from GeneSpring NGS analysis. **B, C, D.** IRAK1 mRNA is reduced following DEK depletion. IRAK1 and DEK mRNA levels were confirmed by TaqMan qRT-PCR to validate RNA-Seq results in three cell lines: UMSCC1, UMSCC47, and UMSCC6 (HPV negative). Experiments were performed twice and standard deviation (SD) depicted. **E.** Western blot analysis confirms IRAK1 protein levels and downstream signaling are also depleted in the absence of DEK. Actin was used as a loading control.

*IRAK1* expression was decreased by 1.4- to 3.2-fold upon DEK loss in HNSCC, as determined by RNA-Seq (Figure 2A). To confirm IRAK1 expression was reduced following DEK depletion, IRAK1 mRNA was independently validated by qRT-PCR in numerous cell lines (Figure 2B–2D). As expected, IRAK1 mRNA levels were reduced in UMSCC1 and UMSCC47 cell lines, as well as in an additional HPV^−^ HNSCC cell line following DEK knockdown. Similarly, DEK knockdown in these cell lines reduced IRAK1 protein expression levels, along with known MAPK signaling, which are downstream targets of IRAK1 (Figure 2E). IRAK repression was also observed in two additional DEK-targeted cell lines UMSCC6 (HPV^−^) and 93VU147T (HPV^+^) (data not shown). Reduced expression of IRAK1 in the absence of DEK was correlated with reduced expression of downstream pathway components, thus suggesting IRAK1 may be a functionally relevant DEK target.

### TCGA data indicates IRAK1 is overexpressed in HNSCC

**Figure 3:**
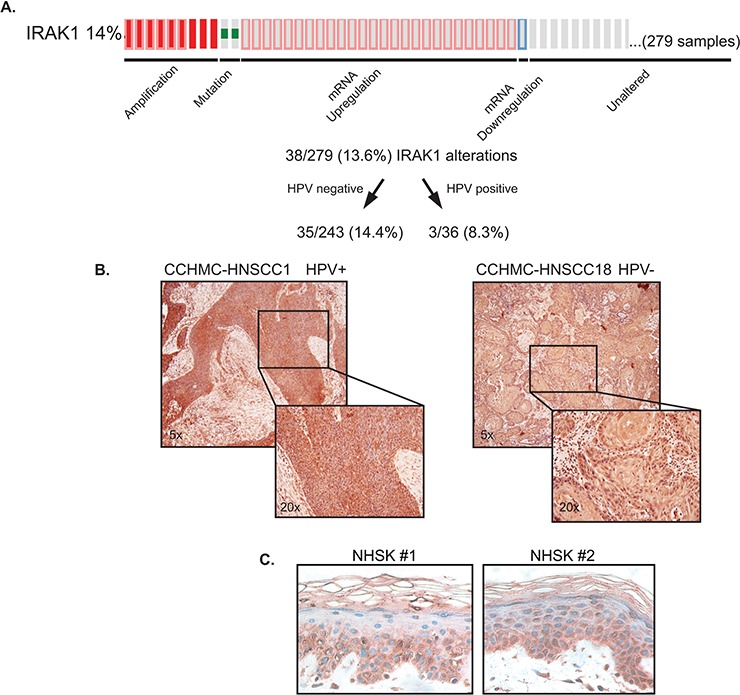
TCGA data indicates IRAK1 is overexpressed in HNSCC **A.** cBioPortal analysis of the TCGA HNSCC database reveals IRAK1 alterations occur in 14% of HNSCC. A total of 279 samples were analyzed and were further broken down into HPV^−^ and HPV^+^ subsets. **B.** IRAK1 is expressed in primary HNSCC tissues. CCHMC-HNSCC1 (HPV+) and CCHMC-HNSC18 (HPV-) were stained for IRAK1 by immunohistochemistry, with intense staining in both the nucleus and cytoplasm. *n* = 4 samples were stained. Images were taken at 5x and 20x magnification. **C.** IRAK1 staining is not expressed in well differentiated layers of normal human skin (NHSK) from unrelated donors. Images were taken at 20x magnification.

Based on the observed IRAK1 transcriptional regulation in HNSCC cell lines, we evaluated publically available TCGA databases to determine whether IRAK1 alterations exist in primary HNSCCs. This data mining revealed *IRAK1* is altered in 14% of HNSCCs, predominantly as a result of gene amplification or mRNA up-regulation (Figure 3A). This overexpression was observed in HPV^+^ and HPV^−^ tumor subsets. To confirm that IRAK1 protein is expressed in HNSCC, we performed immunohistochemistry for IRAK1 on primary HNSCC tissue samples, which were previously described [8]. Examples of HPV^+^ and HPV^−^ specimens are shown (Figure 3B), with strong IRAK1 protein expression detectable in the cytoplasm as expected, and some additional nuclear staining. Since adjacent normal tissue was not present in these specimens, we utilized normal human skin from unrelated donors as a control. IRAK1 staining of 3 specimens (*n* = 3) revealed IRAK1 protein expression was absent from well differentiated layers of human epidermis. Together, this data suggests IRAK1 is highly expressed in HNSCC tumors in line with possible oncogenic activities.

### IRAK1 loss increases apoptosis in HNSCC

**Figure 4:**
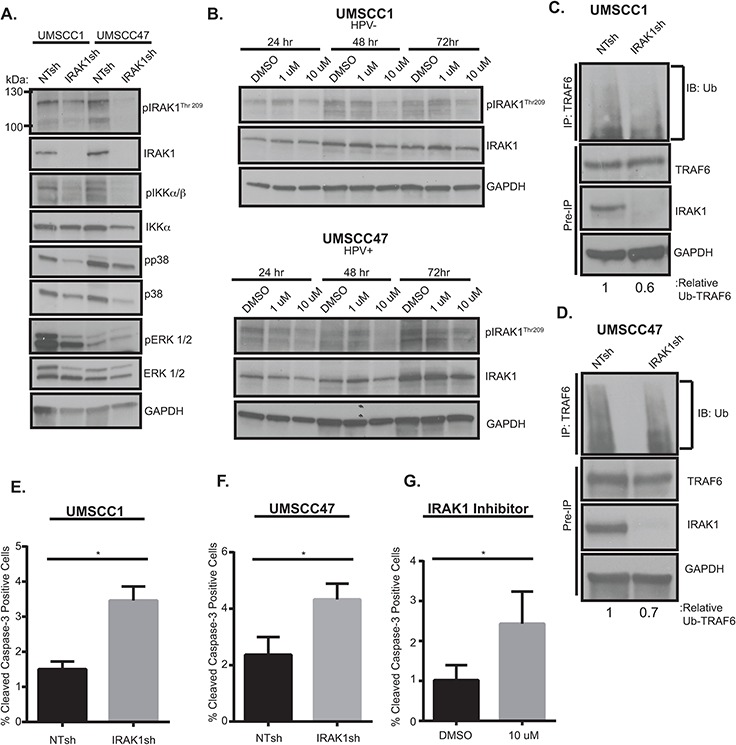
IRAK1 loss increases apoptosis in HNSCC **A.** IRAK1 loss attenuates activation of downstream signaling pathways in HNSCC. UMSCC1 and UMSCC47 were transduced with control (NTsh) or IRAK1 knockdown (IRAK1sh) vectors and protein was collected following selection in puromycin. Whole cell lysates were analyzed by western blot analysis to confirm IRAK1 knockdown, along with reduction in IRAK1 activation (pIRAK1Thr209) and MAPK pathways. GAPDH was used as a loading control. **B.** IRAK1-inhibitor reduces IRAK1 activation in HPV^−^ and HPV^+^ cell lines. UMSCC1 and UMSCC47 cells were plated and DMSO (control) or IRAK1-inhibitor was added the following day at 1 μM or 10 μM final concentrations. Cells were then collected for western blot analysis at indicated time-points. Inhibition of IRAK1 was confirmed by western blot analysis, as measured by phosphorylation of IRAK1. GAPDH was used as a loading control. **C** and **D.** TRAF6 ubiquitination is reduced following IRAK1 loss. Immunoprecipitation was performed on RIPA lysates with the TRAF6 antibody. Western blot was then performed for ubiquitin. Remaining whole cell lysates were analyzed by western blot for TRAF6, IRAK1, and GAPDH. **E** and **F.** IRAK1 loss increases cellular death via apoptosis. Cells were analyzed by flow cytometry for cleaved-caspase 3 conjugated to FITC. Experiments were performed in triplicate with SEM depicted. **G.** IRAK1-inhibitor increases apoptosis. UMSCC1 cells were plated and DMSO or IRAK1-inhibitor was added after cells attached. Cells and media were collected 72 hours later and analyzed for cleaved caspase-3 by flow cytometry. Experiment was performed three times, with SEM depicted. (*=*p* < .05).

Having identified IRAK1 as a candidate effector in HNSCC we next sought to characterize its function by genetic and pharmacologic inhibition. Previously, IRAK1 was proposed to have a tumor suppressive role in HNSCCs [23]. Therefore, we aimed to determine the contribution of IRAK1 to HNSCC phenotypes. We utilized a published IRAK1 shRNA construct to deplete IRAK1 levels in both HNSCC cell lines [20]. IRAK1 knockdown resulted in decreased total and activated IRAK1, as measured by phosphorylation of residue Thr209. In addition, knockdown of IRAK1 coincided with a reduction in in NF-κB (pIKKα/β) and MAPK (p38 and ERK1/2) signaling (Figure 4A), both well-known signaling pathways downstream of activated IRAK1. Chemical inhibition of IRAK1 was also carried out with the IRAK-1/4 inhibitor which has been shown to increase apoptosis in melanoma cells *in vitro* and *in vivo* and to inhibit signaling and cell viability in MDS [20, 24]. This inhibitor is a benzimidazole that is selective for IRAK1 and IRAK4 and shows little specificity for other kinases [24]. Similar to these published studies, 10 μM concentrations of IRAK-1/4 inhibitor attenuated activation of IRAK1 at 24–72 hours post-treatment (Figure 4B) in UMSCC1 and UMSCC47 cells. Activated phospho-IRAK1 complexes with TRAF6, which undergoes Lysine(K)-63 conjugated ubiquitination, a measure of its active state, thereby initiating downstream signaling cascades. To verify that signaling effects observed with IRAK1 loss were a result of a reduction in TRAF6-ubiquitination, TRAF6 was immunoprecipitated and subsequently probed for ubiquitin. TRAF6 ubiquitination was decreased in the absence of IRAK1, suggesting that NF-κB and MAPK signaling is mediated through TRAF6 (Figure 4C-4D). Finally, cellular proliferation and death were assessed upon IRAK1 inhibition to establish a functional role of IRAK1 in HNSCC cells. Significantly increased apoptosis was observed in the absence of IRAK1, either with shRNA or with IRAK1/4-inhibitor (Figure 4E-4G). However, we did not observe any differences in cell cycle profiles (Supplemental Figure 7). These results demonstrate that IRAK1 promotes the survival of HNSCC cells and that IRAK1 inhibition may be a novel therapeutic strategy to enhance cell death in this tumor type.

### IRAK1 and DEK independently regulate HNSCC cellular survival

**Figure 5:**
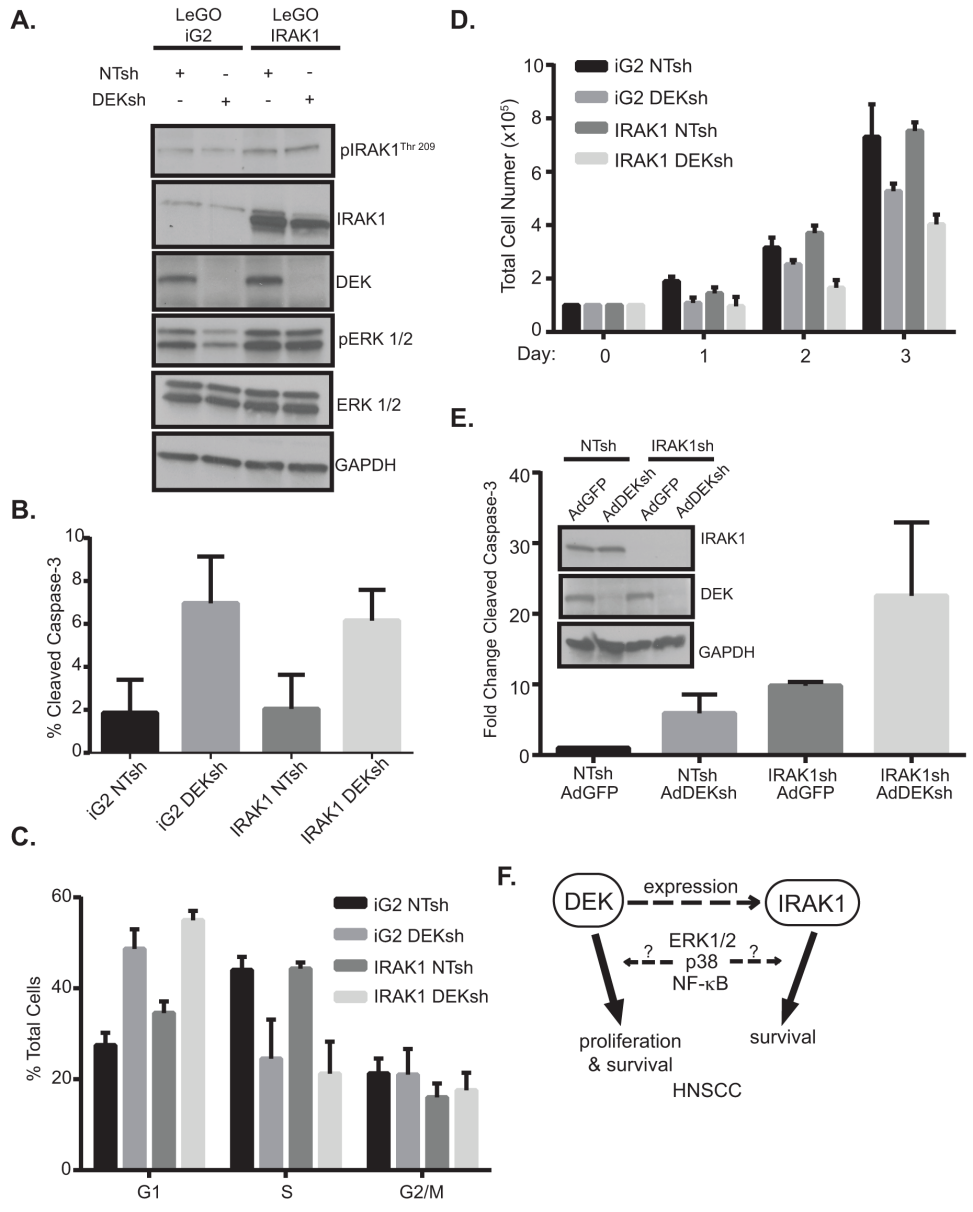
IRAK1 and DEK depletion cooperate to increase apoptosis **A.** IRAK1 overexpression rescues ERK1/2 signaling. Sorted control or IRAK1 overexpressing cells were transduced with control (NTsh) or DEK knockdown (DEKsh) vector. After selection was complete protein was collected and analyzed by western blot. GAPDH was used as a loading control. Growth curves of control (iG2) versus IRAK1 overexpressing cells can be found in Supplementary Figure 8. **B.** IRAK1 and DEK regulate cell growth and viability independently. Cells from (A) were used to analyze apoptosis (B) cellular cycle (C) and cellular proliferation. (D). IRAK1 overexpression did not rescue the phenotypes observed with DEK loss. Experiments were performed twice and SD is represented. **E.** Combined IRAK1 and DEK loss increases cell death. Control and IRAK1 knockdown cells were transduced with control (AdGFP) or DEK knockdown (AdDEKsh) adenovirus. Three days post-adenoviral infection cells were collected and later analyzed for cleaved caspase-3 by flow cytometry. Graph represents fold change compared to NTsh AdGFP samples. Experiments were performed twice and SD represented. **F.** Molecular model. IRAK1 is a novel target in HNSCC. DEK is required for efficient expression of IRAK1 and DEK and IRAK1 also independently contribute to HNSCC cell survival and depletion of either promotes apoptosis and this may be a result of ERK1/2, p38 and/or NF-κB signaling.

To assess if IRAK1 is required for DEK-induced phenotypes in HNSCC, IRAK1 was overexpressed in the presence and absence of DEK (Figure 5A). Interestingly, IRAK1 overexpression rescued phospho-ERK1/2 signaling (Figure 5A), but reconstitution of this pathway was not sufficient to rescue cell death (Figure 5B), cell cycle arrest (Figure 5C), or total cell number (Figure 5D) caused by DEK loss. This observation suggests that DEK and IRAK1 independently contribute to HNSCC cell survival. To determine whether DEK and IRAK1 cooperate to regulate the oncogenic phenotype, and therefore, whether targeting DEK and IRAK1 simultaneously will enhance cell death, we used a dual approach of infecting stably transduced IRAK1 knockdown cells with adenovirus to deplete DEK (AdDEKsh). Either DEK or IRAK1 knockdown alone could induce apoptosis as expected (8–10 fold over control), but the combined effect of IRAK1 shRNA with AdDEKsh infection was greater than that of the respective control cells (20-fold) (Figure 5E). Taken together, these data support a model wherein DEK and IRAK1 function in parallel pathways that control apoptosis, and highlight an additive relationship that may be beneficial for therapeutic intervention (Figure 5F).

## DISCUSSION

A majority of patients with head and neck squamous cell carcinoma present at advanced stages of disease, which contributes to the poor survival outcomes observed. These tumors also notoriously recur, despite aggressive treatment modalities including surgery, chemotherapy and/or radiation therapy, which have frequent side effects that can dramatically and permanently decrease patient quality of life. This suggests these cells have a high proliferative and survival capacity that is necessary for sustained growth of these tumors. Therefore, understanding the relevant targetable mediators of these phenotypes is of the utmost importance. Here we addressed this clinical need by first profiling the transcriptome of HNSCC cell lines that are dependent on the DEK oncogene. DEK is an important regulator of HNSCC growth, and is up-regulated in > 90% of primary HNSCCs tested to date [8]. Although some transcriptional DEK targets have been described, the DEK-dependent transcriptome in squamous cell carcinomas remains unknown. Ontology analysis revealed biological processes significantly altered in the absence of DEK, including adhesion, differentiation, immune regulation, and development. This is in contrast to transcriptional data from neuroendocrine carcinoma of the lung with DEK loss. Shibata *et al*. revealed in their analysis that steroid metabolism, nucleosome assembly, and lipid synthesis and metabolism were altered most often in the absence of DEK [16]. This suggests the effects of DEK loss in malignancies are cell-type dependent.

Here we have identified DEK-dependent gene expression that supports phenotypes previously defined, along with new systems of interest for future studies. Although DEK overexpression is published to promote the migration and invasion of breast and HNSCC cells, alterations in cellular adhesion have not been pursued [25]. Our data suggests this may be one mechanism by which DEK promotes invasion (Figure 1C). Furthermore, we have correlated the DEK-dependent transcriptome with common transcription factor binding sites that are associated with DEK loss (Supplementary Tables 1–3). Many notable transcription factor binding sites and known targets of DEK were identified, including p53, CEBP, and p65 validating the importance of DEK in multiple cancer types [15, 26]. For example, DEK-dependent control of p53 binding sites was expected given that DEK loss was reported to lead to the stabilization of p53 [21]. This correlated with the up-regulated induction of several p53 regulated genes including *BCL11B*, *KRT15*, and *PIM1*. Herein we focused on the regulation of genes with roles in immune cell signaling. These included NF-κB and MAPK driven genes such as MAPK14, TNFAIP3, IL6, and IRAK1. We chose to probe the role of the IRAK1 serine/threonine kinase, a driver of inflammatory pathways in hematological disease, based on its function as a central signaling hub in the cytoplasm, and as a targetable molecule in MDS and AML.

The role of IRAK1 in solid tumors has not been explored extensively, but our data suggest oncogenic and potentially targetable activities in HNSCCs. IRAK1 was transcriptionally up-regulated and amplified in a proportion of HNSCCs in the TCGA, in line with a newly discovered functional requirement for maximal survival of HPV positive and negative HNSCC cell lines. Such a role was uncovered through IRAK1 knockdown using stable lentiviral vectors, as well as through chemical inhibition. Downstream IRAK1 signaling was suppressed through TRAF6, attenuating activation of NF-κB and MAPK and stimulating cancer cell death, thus highlighting the potential use of IRAK1 inhibitors in the treatment of HNSCC.

Our data identified IRAK1 as a component of the DEK-dependent transcriptome whose expression in HNSCC contributes to tumor cell survival. IRAK1 drives ERK1/2 signaling, but this alone was not sufficient to rescue cell growth in the absence of DEK. This is not surprising given the large network of genes regulated by DEK and suggests multiple genes are required to maintain a proliferative state. It is also possible that ERK1/2 signaling may be unimportant in, or may modify the response to DEK loss. Finally, we investigated a possible effect of DEK loss on NFκB signaling. NFκB can also be activated downstream from the IRAK1 cascade. Western blot data from both cell lines demonstrate an unexpected reduction in total IkBα protein (Supplementary Figure 9). Together, these data suggest DEK/IRAK targeting may in fact activate the NFκB pathway, and highlight unexpected signaling connections between DEK/IRAK1 and NFκB in HNSCC which remain to be defined.

Although IRAK1 was not by itself sufficient to rescue DEK-deficiencies, the combined targeting of DEK and IRAK1 demonstrated an additive relationship. This additive effect emphasizes the large network of signaling hubs through which DEK functions, independent of IRAK1, which can be further extracted from the transcriptional data and explored in future experiments. Importantly, this work has defined IRAK1 as one functionally important driver of HNSCC survival. Interestingly, tumor suppressive functions of IRAK1 have been proposed in a recent publication in oral squamous cell carcinoma cells (OSCC). Hung *et al*. described miR-146a, a known regulator of IRAK1, as overexpressed in OSCC. Other publications define miR-146a as tumor suppressive, where its loss hyper-activates IRAK1 and may be one mechanism for IRAK1 overexpression [27]. In OSCC, exogenous miR-146a expression increased orthotopic tumors and metastasis of SAS cells and reduced IRAK1 protein levels. In these same cells, IRAK1 knockdown combined with TRAF6 knockdown by siRNA increased invasion and tumor volume, but IRAK1 knockdown alone had few effects [23]. Here, we utilized oropharyngeal HNSCC cells lines wherein IRAK1 surprisingly exhibited oncogenic functions. These opposing findings may be related to the site of origin for each cell line. Additionally, TCGA data wherein IRAK1 is largely overexpressed in HNSCCs supports the hypothesis that IRAK1 contributes to oncogenic phenotypes [23]. Many possibilities exist to explain these discrepancies. IRAK1 functions may be anatomically or cell line dependent, or the method of inhibition may be important (acute (siRNA) versus stable (shRNA)). Additionally, IRAK1 expression may be a double-edged sword and careful balance of its expression might be required. TCGA data in other squamous tumors, such as cervical cancer, identified one patient with homozygous deletion of *IRAK1* and two with truncating mutations. The other twenty specimens where IRAK1 was altered were due to copy number amplification and mRNA up-regulation. Overexpression of IRAK would then be a predictive marker of optimal response to IRAK1 inhibitors and may be a fruitful biomarker across various types of malignancies.

## MATERIALS AND METHODS

### Cell culture

HPV negative UMSCC1 and UMSCC6, and HPV positive UMSCC47 head and neck cancer cell lines were cultured in DMEM (Gibco, New York, NY, USA) supplemented with 1% hydrocortisone (HPV^−^ only), 10% fetal bovine serum, antibiotics and antifungals.

### Lentiviral transduction

Cell lines were transduced with lentiviral pLKO.1 vectors for either nontargeting control shRNA (NTsh), IRAK1sh (TRCN0000000543, OpenBiosystems, Lafayette, CO, USA), or DEK832 (DEKsh, Sigma-Aldrich Mission shRNA library, St Louis, MO, USA) in the presence of polybrene (8 μg/mL). Cells were selected in puromycin at a final concentration of 1 μg/mL.

### Adenoviral transduction

Cells transduced with control (NTsh) and IRAK1 knockdown (IRAK1sh) lentiviruses were plated in equal densities and kept under puromycin selection. 48 hours post-plating cells were transduced with control (AdGFP) or DEK knockdown (AdDEKsh) adenoviral vectors at 10 infectious units per cell as previously published [21]. 72 hours post-transduction, cells and media were collected and fixed to analyze for flow cytometry (see below). IRAK1 and DEK knockdown were confirmed by western blot analysis.

### IRAK1 overexpression

UMSCC1 cells were lentivirally transduced with control (LeGo-iG2) or IRAK1 overexpression (LeGO-IRAK1) vectors in the presence of 8 ug/mL polybrene. Cells were sorted based on GFP-positivity and expanded for experiments post-sorting. IRAK1 overexpression did not alter the growth of these cells (Supplementary Figure 8). Creation of these vectors has been described previously [28].

### cBioPortal analysis

The results depicted here are in whole or part based upon data generated by the TCGA Research Network: http://cancergenome.nih.gov [29, 30]. For IRAK1 expression z-score thresholds were set at 2.0.

### RNA-sequencing

Transduced and selected UMSCC1 and UMSCC47 NTsh and DEKsh cells were collected and processed with a ZR RNA MiniPrep kit (R1064, Zymo Research, Irvine, CA, USA), per kit instructions. A portion of the final RNA isolate for each sample was submitted for quality assurance prior to RNA-Sequencing. RNA-Sequencing was performed by the CCHMC DNA Sequencing and Genotyping Core on an Illumina HiSeq2500 for single-end sequencing with 50 base pair reads. The data discussed in this publication have been deposited in NCBI's Gene Expression Omnibus [31] and are accessible through GEO Series accession number GSE70462 (http://www.ncbi.nlm.nih.gov/geo/query/acc.cgi?acc=GSE70462).

### GeneSpring NGS analysis

RNA-Seq files were imported into GeneSpring Multi-Omic Analysis Software V12.6 (Agilent, Santa Clara, CA, USA) and sequences were aligned to the reference genome, hg19/GRCh37, which efficiently aligns reads spanning known or novel splice junctions. The reference annotations were produced by the Ensembl project [32]. Aligned reads were filtered on base quality, with a quality threshold >=30. The aligned gene read counts were quantified and used to compute reads per kilobase per million reads (RPKMs) for each transcript in each sample. Raw counts were normalized using the DESeq algorithm and threshold set to 1. Subsequent filtrations removed all genes with fewer than 3 reads in each sample. Fold change was calculated as DEKsh/NTsh with a cut-off of 1.4 fold change. Venn diagrams were created in GeneSpring and entity lists were translated from UMSCC1. Gene lists were submitted to ToppGene (http://toppgene.cchmc.org) for functional enrichment analysis [33].

### Network analysis

All of the network analyses and visualizations were performed using the NetWalker software and were described in detail previously [34].

### Transcription factor binding site analysis

In order to identify enriched (*p* value <0.05) putative transcription factor binding sites within the up- and down-regulated genes, we mined the catalog of human, mouse, rat, and dog conserved regulatory motifs in promoters using the ToppGene server [33, 35].

### Quantitative RT-PCR

RNA was collected with Trizol (Invitrogen, Grand Island, NY, USA) and reverse transcribed to cDNA using the QuantiTect Reverse Transcription kit (Qiagen, Valencia, CA, USA). cDNA expression was detected with TaqMan Gene Expression Master Mix and probes (Applied Biosystems). Data was analyzed using the ΔΔCt method and values calculated relative to GAPDH. TaqMan probes were as follows: DEK (Hs01078267_m1), IRAK1 (Hs01018347_m1), and GAPDH (Hs02758991_g1).

### Western blotting

Whole cell lysates were harvested using Laemmli buffer and a total of 20 μg of protein was analyzed as described previously [21]. Membranes were probed with DEK (1:1000, BD Biosciences, San Jose, CA, USA), IRAK1 (1:1000, Santa Cruz Biotechnology, Dallas, TX, USA (sc-7883)), TRAF6 (1:1000, Santa Cruz (sc-7221)), phospho-IRAK1 (Thr209, 1:800, Assay Biotechnology Company (A1074), Sunnyvale, CA, USA), phospho-p38 (1:500, Cell Signaling Technologies (4361)), p38 (1:1000, Cell Signaling Technologies (9212)), phospho-ERK1/2 (1:1000, Cell Signaling Technologies (4377)), ERK1/2 (1:1000, Cell Signaling Technologies (4695)), phospho-IKKα/β(1:500, Cell Signaling Technologies (2697)), IKKα(1:1000, Cell Signaling Technologies (2682)), phospho-IκBα (1:500, Cell Signaling Technologies (9246)), IκBα (1:1000, Cell Signaling Technologies (4812)), α-tubulin-HRP (1:10,000 Cell Signaling Technologies (9099)), actin (1:10,000 a gift from James Lessard), and GAPDH (1:1000, Cell Signaling Technologies (5174)).

### Flow cytometry for cleaved caspase-3

Lentivirally transduced cells were plated at equal densities and collected 48 hours later. Cells were fixed and prepped following the BD FITC Active Caspase-3 Apoptosis kit protocol (BD Biosciences). Adenovirally transduced cells were prepped with an Alexa-Fluor 647 conjugated cleaved-caspase 3 antibody to account for GFP-positivity (Cell Signaling Technologies (9602)). Analysis was performed on a BD FacsCanto and data analyzed on FlowJo software (Tree Star, Ashland, OR, USA). Experiments were performed 3 times with standard error of the mean (SEM) represented.

### Flow cytometry for cell cycle analysis

Lentivirally transduced cells were plated in equal numbers and 48 hours later were pulsed with 10 μM BrdU for 45 minutes. Cells were collected and prepped following the BD Pharmigen APC BrdU flow kit and analyzed on a BD FacsCanto. Data was analyzed on FlowJo Software as above. Experiments were performed 3 times and SEM represented.

### IRAK1 inhibitor

UMSCC1 cells were plated at equal densities and IRAK1 inhibitor (IRAK-1/4 inhibitor, I5409, Sigma-Aldrich, St. Louis, MO, USA) added the same day for caspase-3 flow cytometry experiments. DMSO was added in equal volume for a control. Cells and media were collected 72 hours later and prepared and analyzed as above. For the time-course experiment, cells were plated and inhibitor was added the following day, with protein collected at 24, 48, and 72 hours and analyzed by western blot for IRAK1 inhibition.

### Immunohistochemistry

The IRB-approved collection of primary human tumor tissue specimens and immunohistochemistry staining protocol were previously described [8]. Sections from *n* = 4 primary tumor specimens and *n* = 3 normal human skin samples were probed with IRAK1 antibody (1:50, sc-7883, Santa Cruz). Skin samples were obtained from consented donors at Cincinnati Children's Hospital Medical Center in accordance with an approved IRB protocol. Images were captured with a Leica DM2500 microscope and LAS software (Leica Microsystems Inc., Buffalo Grove, IL, USA) at the indicated magnifications.

### TRAF6 immunoprecipitation

Samples were lysed using RIPA buffer containing protease and phosphatase inhibitors and protein concentration determined using BCA Protein Assay Kit (Pierce 23225). 600 μg of each sample was used to perform TRAF6 immunoprecipitation. Lysates were incubated with A/G beads (sc-2003, Santa Cruz, Dallas, TX, USA) and incubated with TRAF6 antibody (sc-7221, Santa Cruz). Samples were loaded onto a 4–15% gradient Mini-PROTEAN TGX Precast Gel (BioRad, Hercules, CA, USA) and proteins separated by SDS-PAGE electrophoresis. Membranes were probed with ubiquitin primary antibody (sc-8017, Santa Cruz). Protein from the original lysis, prior to immunoprecipitation, was run following the above western blot protocol. Membranes were probed with IRAK1, TRAF6, and GAPDH. Densitometry was performed using ImageJ software.

### Growth curves

Control and IRAK1 overexpressing cells were plated at equal densities, in triplicate, and total cell number counted over 3 days. Experiments were performed twice with SD represented.

### Statistics

Statistical analysis was performed using GraphPad Prism 6 software (La Jolla, CA, USA). Student's *t*-test was used to calculate *p*-values, where *=*p* ≤ .05 and **=*p* ≤ .01.

## SUPPLEMENTARY TABLES AND FIGURES


