# Supplementary material for: IRAK1 is a novel DEK transcriptional target and is essential for head and neck cancer cell survival
Source: Oncotarget. 2015 Oct 26;6(41):43395–407. doi: 10.18632/oncotarget.6028 (PMC4791239; doi:10.18632/oncotarget.6028)
Supplement: Supplementary file 1 [file oncotarget-06-43395-s001.pdf]

SUPPLEMENTARY TABLES AND FIGURES

Supplementary Table S1: Transcription factor binding site (TFBS) analysis for genes up-regulated in UMSCC1 and UMSCC47

TF binding site ID	log *p*-value	Up-regulated Genes
V$P53_DECAMER_Q2	2.972	ABCA12, AKAP6, ARHGEF37, ARHGEF6, BCL11B, DOCK9, FLRT1, GJB4, HCAR2, HOXB3, KRT15, LRP1, PIM1, PITPNC1, PPL, PRDM12, PTAFR, PTPRG, RAP2B, RARB, ST5, TBC1D8, TECTA
V$E12_Q6	2.610	ABLIM3, ALDH1A2, ARVCF, CAMK2A, COL4A3, CORO2B, CPZ, CRELD1, GFAP, GJB2, HIF3A, ITGB4, MDGA1, MYH14, NFATC4, NPEPPS, POU2F3, SASH1, SELL, SORCS2, SPTB
RTAAACA_V$FREAC2_01	2.452	ABCA1, ABLIM3, ACVR1C, ADAMTS13, ARHGEF6, ARID4A, ASAH1, BACH2, BCL11B, BIK, C1QTNF3, CCND1, CDKN1B, DAPP1, DOCK3, EMP1, EPHB3, FBXW7, GATS, GCNT2, GPR110, GPR4, GRK5, HCAR2, HCAR3, HOXA5, HSPG2, IL16, L3MBTL1, LEMD1, LRP1, MAML3, MGAT4B, MMP13, MN1, NEBL, NGEF, NLGN3, NR4A2, PBXIP1, PCDHGA5, PHEX, PIK3IP1, PITPNC1, PLAG1, PLAU, PPAP2B, PRDM1, PROX1, RP1, RPS6KA5, SDCBP2, SLC34A3, SLC4A1, SOCS1, SPRY4, TCF7L2
V$TAL1BETAE47_01	2.110	ABHD16A, ASB4, DTX2, HOXB6, HRK, KCNIP2, NPR2, OMG, PBXIP1, PCDH1, PCDH12, PHEX, PLXNA3, PPAP2B, PYY, RCAN2, RNF19A, SCN1B, UPK2, WNT9A
V$IRF_Q6	1.932	ACSL5, ARHGEF6, CCND1, DAPP1, ESR1, HLA-C, HLA-F, HOXB3, MLLT3, MUSK, NCF1, NPEPPS, PLXNC1, PSMB10, SELL, SOCS1, TCF7L2, ZBP1
V$AREB6_01	1.876	CDKN1B, CEL, CRELD1, CSAD, CYP26B1, FBXO24, FXYD3, GAD1, HOXB6, IQGAP2, MAP3K5, PCDHGA5, PLAG1, PROX1, RRAD, S100A9, SLC4A11, STRC, STX1A, VWF
KRCTCNNNNMANAGC_ UNKNOWN	1.816	HIST1H1C, HIST1H2AC, HIST1H2AJ, HIST1H2BD, HIST1H2BJ, HIST2H2AC, HIST2H2BE, HIST3H2BB
V$P53_02	1.809	ABCA12, ACSL5, AKAP6, ASIC4, BCL11B, FLRT1, GAD1, GCNT2, HCAR2, KRT15, NR4A3, PIK3R2, PITPNC1, PPL, PRDM12, PTPRG, RARB, ST5, TMC7
TGANNYRGCA_ V$TCF11MAFG_01	1.770	ABCC6, ANGPTL4, ATL1, BCL6, CBX6, CLCN5, CPZ, DRP2, DTX2, ESR1, FBLN2, LRP1, NR4A3, OMG, PFKFB1, PIM1, PRDM1, RAG1, SLC22A18, TFEC, TREML2
TGTTTGY_V$HNF3_Q6	1.760	ALDH1A2, ARHGEF6, ASB4, BCL11B, BCL6, CBX6, CDH12, CNTNAP2, CSAD, DOCK9, DTX2, EFNA1, ESR1, FCGBP, FLRT1, GJB4, HCAR1, HCAR2, HCAR3, HIST1H1C, HIST1H2BD, HOXB3, HS3ST5, IL16, IQGAP2, ITM2B, MAST4, MGAT4B, MUSK, NFATC4, NOS3, NR4A3, OMG, PDGFRA, PHEX, PRDM1, PTPRG, SHC3, SLC4A11, SNCG, SPAG8, ST5, TCF7L2, TFEC

(*Continued*)

TF binding site ID	log *p*-value	Up-regulated Genes
V$AML1_01	1.701	ACSL5, AKAP3, ANXA8L2, CD6, EMP1, GRK5, MMP13, MMP14, MPL, MR1, PDZK1, PLXNC1, RAG1, S100A9, SCEL, SLC37A2, STRC, TLL2
V$AML1_Q6	1.701	ACSL5, AKAP3, ANXA8L2, CD6, EMP1, GRK5, MMP13, MMP14, MPL, MR1, PDZK1, PLXNC1, RAG1, S100A9, SCEL, SLC37A2, STRC, TLL2
YATGNWAAT_V$OCT_C	1.680	ACBD4, ALDH1A2, ARHGAP4, CCND1, DNAH5, EPHB3, FBXO24, GPR4, HIST1H2AC, HIST2H2AC, HIST2H2BE, HOXA5, HOXB6, MMP17, NR4A3, PFKFB1, PIWIL4, POU2F3, PRDM1, RARB, RNASE4, SASH1, SEMA6C, SH3BGRL, WNT9A
V$PPARA_02	1.644	AHCYL2, CBX6, CDH17, EPHB1, HOXA5, MNT, NLGN3, PSCA, RTN2, SERPINF2, UCN2
V$PPARA_01	1.640	CD36, PAPLN, PDZK1, PTPRG, ZNF547
V$FREAC4_01	1.603	ADAMTS13, ALDH1A2, EMP1, HSPG2, MAST4, PHEX, PRDM1, PTAFR, SLC34A3, SPRY4, TCF7L2, TECTA
RYCACNNRNNRNCAG_UNKNOWN	1.543	AHCYL2, FYB, IL17RE, MB, PBXIP1, RHOF, UPK2
V$NFKAPPAB65_01	1.480	COL11A2, CSF1R, CYP2D6, DSC2, GNG4, GRK5, KCNN2, KRT23, PCDH12, POU2F3, PRDM12, PTGES, SDC4, TCEA2, TRIB2, TSLP, ZMYND15
V$GATA_Q6	1.475	ABCA12, COL4A3, DENND1B, EPB42, FMO1, GPR116, HOXB6, HS3ST5, PDGFRA, PFKFB1, PLAG1, PVRL4, SLC4A1, SPRY4, SYT7
TGCCAAR_V$NF1_Q6	1.470	ABCA9, ABLIM2, AGER, AQP11, ARHGEF6, BACH2, BCL6, C1QTNF3, C4A, C4B, C6orf223, CLCN5, COL4A3, DDO, EIF4EBP3, ESR1, FLRT1, GCNT2, GFAP, GNAT2, IL16, INHBC, MAST4, MPL, MUSK, NAPB, NLGN3, PAPLN, PAQR6, PBXIP1, PHEX, PLXNC1, PROX1, RARB, RPS6KA5, RRAD, SPRY4, SYTL2, TSLP, VNN2, XDH
V$PAX4_03	1.442	AGER, ANXA4, CBX6, COL11A2, FBXO24, HIF3A, HOXB6, KCNN2, LRP1, MMP14, MNT, MPL, NFATC4, NLGN3, PPAP2B, PRDM1, SERPINI1, TRIM46
RTTTNNNYTGGM_UNKNOWN	1.390	ALDH1A2, ESR1, FHIT, LRP1, MARCO, MAST4, NOS3, PHEX, PTPRCAP, PTPRG, STX6, TECTA
TGACATY_UNKNOWN	1.376	ACVR1C, AKAP3, AOC2, ARHGAP4, ARHGEF6, ASB11, ASB4, CLCN5, DSC2, FAM71F1, FBXO32, HOXB3, ITM2B, KCNN2, LEMD1, LRP1, MLLT3, MNT, NEBL, NFATC4, PBXIP1, PDGFRA, PITPNC1, PLAG1, PRDM1, PTPRC, RAPSN, RARB, RCAN2, RNF19A, RRAD, SASH1, SH3BGR, SLC4A1, ST5, STX6, TBC1D8, TECTA
V$CEBP_01	1.358	BCL11B, CLDN16, CSAD, CYP2D6, DOCK9, EVI2A, GCNT2, GSTA4, HOXB3, HOXB6, MNT, NFATC4, PFKFB1, PLXNC1, PRDM1, RNF19A, S100A9, ST5

(*Continued*)

TF binding site ID	log *p*-value	Up-regulated Genes
V$OCT_C	1.358	ARHGAP4, EPHB3, FBXO24, GCNT2, GPR4, HIST1H2AC, HIST2H2AC, HIST3H2BB, HOXB3, MMP17, PFKFB1, POU2F3, PRDM1, SASH1, SEMA6C, SH3BGRL, STAT4, TLL2
GATAAGR_V$GATA_C	1.336	ABCA12, ADPRHL1, EPB42, FCGBP, FMO1, HS3ST5, HYAL3, KRT15, KRT23, LEMD1, MLLT3, MST1, NEBL, PFKFB1, PLAG1, PTPRC, SLC34A3, SLC4A1, SPRY4, SYT7
V$COREBINDINGFACTOR_Q6	1.326	ADD1, ANXA8L2, B3GNT5, CD6, CYGB, EMP1, EVI2A, HOXB6, IL17RE, MMP13, MPL, NR4A3, PDZK1, PITPNC1, RAG1, S100A9, SLC37A2, STRC
CCANNAGRKGGC_ UNKNOWN	1.323	ACBD4, GAD1, GRK5, LRP1, MB, PCDH12, POU2F3, SORCS2, UPK2
V$HNF4_Q6	1.307	ABCA12, ARHGEF37, C4A, C4B, EMP1, FBXW7, HOXA5, PDZK1, PRDM1, PROX1, PTPRH, RARB, SLC5A2, SLPI, SP140, SPINT1, SULT2B1

Enriched TFBS were found using the ToppGene server

Supplementary Table S2: Transcription factor binding site analysis for genes down-regulated in UMSCC1 and UMSCC47

TF binding site ID	log *p*-value	Down-regulated Genes
V$SRF_C	3.170	ACVR1, CYR61, DIXDC1, EMILIN2, HOXA3, MAPK14, MYL9, NR2F2, PPP2R3A, RAB30, SCOC, TAGLN, THBS1, TNNC1, ZAK
V$SRF_Q4	2.907	DIXDC1, EDN1, EMILIN2, HOXA3, MAPK14, MYL9, NR2F2, PPP2R3A, RAB30, SCOC, TAGLN, THBS1, TNNC1, VGF, ZAK
CTTTAAR_UNKNOWN	2.802	ANKRD28, ATP5SL, AXL, CREB3L1, CYR61, DCX, DOCK11, EDN1, EGLN3, ETHE1, FPGT, GAN, GNG11, GNGT1, HOXA3, HOXA4, HPS3, KCTD8, KIRREL3, KTN1, MAPKAPK3, NELL2, NR2F2, NR5A2, PDE3B, PLXDC2, PPM1E, PPP2R3A, PXK, RBM24, SCD, SCML1, SMARCA1, SOCS2, STEAP2, TAGLN, TBX2, TCF4, TGFB2, VGF, ZNF593
TGGAAA_V$NFAT_Q4_01	2.689	ANKRD28, ANTXR1, APOM, ARHGEF25, CALB1, CCNI, CD86, CDC42EP3, CKS1B, CNIH1, COL2A1, CXCL10, CYR61, DNMT3B, EFNB3, ERBB4, FAM155B, FZD7, GDA, GFOD1, GPR150, HOXA13, HOXA3, HOXA4, HSPH1, HTR7, IL6, IL7R, KCNQ5, KIRREL3, LRRC2, MGP, MTX2, NGF, NINJ2, NR5A2, NRAS, PAK1IP1, PDE3B, PIGW, POLG2, PPP2CA, PPP2R3A, RAB30, RCN3, RNF128, SCRN3, SKP2, SLA, SLC43A1, SMYD2, SNX12, SOCS2, STEAP2, SV2A, TBX2, TCF12, TCF4, TGFB2, TJP2, TNFRSF11B, TUB, VEGFC, VGF, XPNPEP1, ZIC5, ZNF593
CCAWWNAAGG_V$SRF_Q4	2.622	DIXDC1, RAB30, SCOC, TAGLN, TCF4, THBS1, TNNC1, ZAK
V$COUP_01	2.506	APOM, CKS1B, CNIH1, DOCK11, EFNB3, FAM155B, HOXA3, IL21R, NR2F2, NYAP1, PDE3B, PPP2R3A, RPL34, SMYD5, STEAP2
V$ER_Q6_02	2.506	ADAMTS15, CAPN12, CD37, DACT2, DCX, FHL1, HOXA3, HPCAL4, KIRREL3, MTX2, NR2F6, PPM1E, SMARCA1, STEAP2, TOMM40
V$EN1_01	2.073	ERBB4, FHL1, HOXA4, HTR7, MAPK14, NRAS, PEX2, TCF4
V$MYC_Q2	2.068	CA14, COL2A1, HPCAL4, LDHA, NRAS, PFDN2, SC5D, SHMT2, SLC43A1, TGFB2, ZNF593
V$NMYC_01	1.978	ANGPT2, BCL2, CA14, COL2A1, GJA1, MAPKAPK3, NRAS, RAB30, RNF128, SC5D, SLC43A1, SOCS5, VGF, ZCCHC7
V$OCT1_01	1.978	CDC42EP3, CYR61, EGLN3, HOXA3, HPCAL4, IRAK1, LHX6, NR2F2, PPP2R3A, PYGO1, SLC19A3, SLC7A11, TCF12, TCF4
RYTGCNNRGNAAC_V$MIF1_01	1.826	BTG4, EFNB3, HMGCS1, HSF1, LHX6, ZNF593
V$SMAD_Q6	1.813	BCL2, CCNI, CKS1B, GARNL3, KCNQ5, LHX6, NYAP1, PRX, SMARCA1, SMYD2, SNX12, SYT11, TAGLN
V$NFAT_Q4_01	1.690	ANKRD28, HOXA3, KCNQ5, KIRREL3, MRPL30, NR5A2, PAK1IP1, PDE3B, PPP2R3A, RNF128, SCRN3, SLA, TJP2
V$NRF2_01	1.680	BCL2, C11orf84, CKS1B, E2F4, FBXO22, HSPH1, NRAS, PAFAH1B2, RPL34, TIMM8B, TOMM40, ZNF22
CACGTG_V$MYC_Q2	1.618	ANGPT2, BEX2, CA14, COL2A1, DZIP1, GAR1, GJA1, HOXA3, HOXA4, HPCAL4, HPS3, HSPH1, KCNQ5, KIAA1033, LDHA, MAPKAPK3, NPTX1, NRAS, PDP2, PDPR, PFDN2, PIGW, RAB30, RNF128, RRS1, SC5D, SHMT2, SLC43A1, SOCS2, SOCS5, TBC1D5, TCF4, TGFB2, VGF, ZCCHC7, ZNF593

(*Continued*)

TF binding site ID	log *p*-value	Down-regulated Genes
V$MYCMAX_02	1.603	ANGPT2, APLN, COL2A1, DZIP1, HOXA3, HSPH1, KCNQ5, LRRC2, RAB30, RNF128, TCF4, TGFB2, VGF
AAGWWRNYGGC_UNKNOWN	1.583	ANKRD28, HOXC10, IRAK1, NRAS, PPM1E, RFC1, SMYD5
TAAWWATAG_V$RSRFC4_Q2	1.577	BEX2, GRB14, HOXA4, KCNQ5, KTN1, PPP2R3A, SV2A, TCF4, TNNC1
V$CEBP_Q3	1.516	CYR61, DYRK3, EFNA5, GPLD1, NR2F2, PPM1E, RAB30, SNX12, TCF12, TGFB2, WNT10B, ZAK
V$RSRFC4_Q2	1.501	ADAM11, BNIP3, CDC42EP3, KCNQ5, KTN1, MRPS23, PTPN1, SCML1, SLCO2A1, SV2A, TNNC1
V$POU3F2_02	1.460	BBX, CYR61, DIXDC1, HOXA3, HOXA4, HPCAL4, IRAK1, LHX6, NRAS, OLFML2B, PAK1IP1, PDE3B
V$STAT5A_03	1.460	BBX, GPR150, HOXA3, HOXA4, HOXC10, MAPK14, PDE3B, PLXDC2, PPP2R3A, SV2A, TCF4, TNFRSF11B
V$MEF2_Q6_01	1.460	ADAM11, ANGPT2, CDC42EP3, GAN, GRB14, KCNQ5, KTN1, LRRC2, PRX, SMARCA1, SV2A, TNNC1
V$ELK1_02	1.457	CKS1B, DDIAS, FBXO22, MTX2, NRAS, OGG1, RFC4, TIMM8B, TOMM40, TRO, ZNF22
WWTAAGGC_UNKNOWN	1.456	BBX, DCX, ETHE1, IRAK1, MYL9, NR2F2, NRAS, TBX2
V$CEBP_Q2	1.442	EDN2, LHX6, MAPK14, NR2F2, PDE3B, PTX3, PYGO1, RRM2B, SKP2, TCF12, TCF4
GAANYNYGACNY_UNKNOWN	1.430	CKS1B, GDA, NR2F2, SUMO1, TCF12
V$FOXD3_01	1.428	ANKRD28, CYR61, DIXDC1, HOXA3, KCNQ5, NRAS, PRICKLE2, RNF128, SLC39A8, TGFB2
V$SRF_Q6	1.420	DIXDC1, EMILIN2, HOXA3, LDHA, MYL9, NR2F2, PPP2R3A, SCOC, TAGLN, THBS1, TNNC1, ZAK
V$OCT1_05	1.394	BCL2, EDN1, HOXA3, IL6, LHX6, NR2E1, PPM1E, PPP2R3A, SCML1, SLC19A3, SLC7A11, TCF12
V$USF_Q6_01	1.372	APLN, HOXA3, KIAA1033, NPTX1, NRAS, PDP2, PFDN2, SC5D, SLC43A1, SOCS5, VGF
V$ATF1_Q6	1.372	CCNI, CD37, DOK1, DYRK3, HOXA4, HOXC10, KCTD8, LDHA, NR2E1, RRM2B, VGF
V$CP2_02	1.358	CDC42EP3, DCX, DDIT4, FOXL2, RAB30, RNF121, TBC1D5, TBX2, TNNC1, WNT10B, XPNPEP1
V$USF_02	1.342	APLN, CA14, CYR61, FAM155B, NPTX1, NYAP1, PFDN2, RFC1, RNF128, TCF12, TGFB2, UBE2L6
V$SRF_Q5_01	1.331	DIXDC1, EMILIN2, MAPK14, MYL9, NR2F2, NRAS, PPP2R3A, SCOC, TAGLN, THBS1, ZAK
V$IK1_01	1.317	CD86, IL6, LHX6, MMP16, NEK4, NR2F6, RNF128, SMYD2, SOCS2, TNFSF18, VGF, ZIC5

Enriched TFBS were found using ToppGene server

Supplementary Table S3: Transcription factor binding site analysis for genes differentially expressed in both UMSCC1 and UMSCC47, but oppositely regulated (up vs. down)

TF binding site ID	log *p*-value	Oppositely-Regulated Genes
V$GATA6_01	1.567	BCL6, BTG2, CLEC18C, COL4A3, DENND1B, FMO1, HOXB3, HOXB6, HS3ST5, KRT15, KRT23, PDGFRA, PFKFB1, PTPRG, PYY, RNF112, SPRY4, SYT7

Enriched TFBS were found using ToppGene server

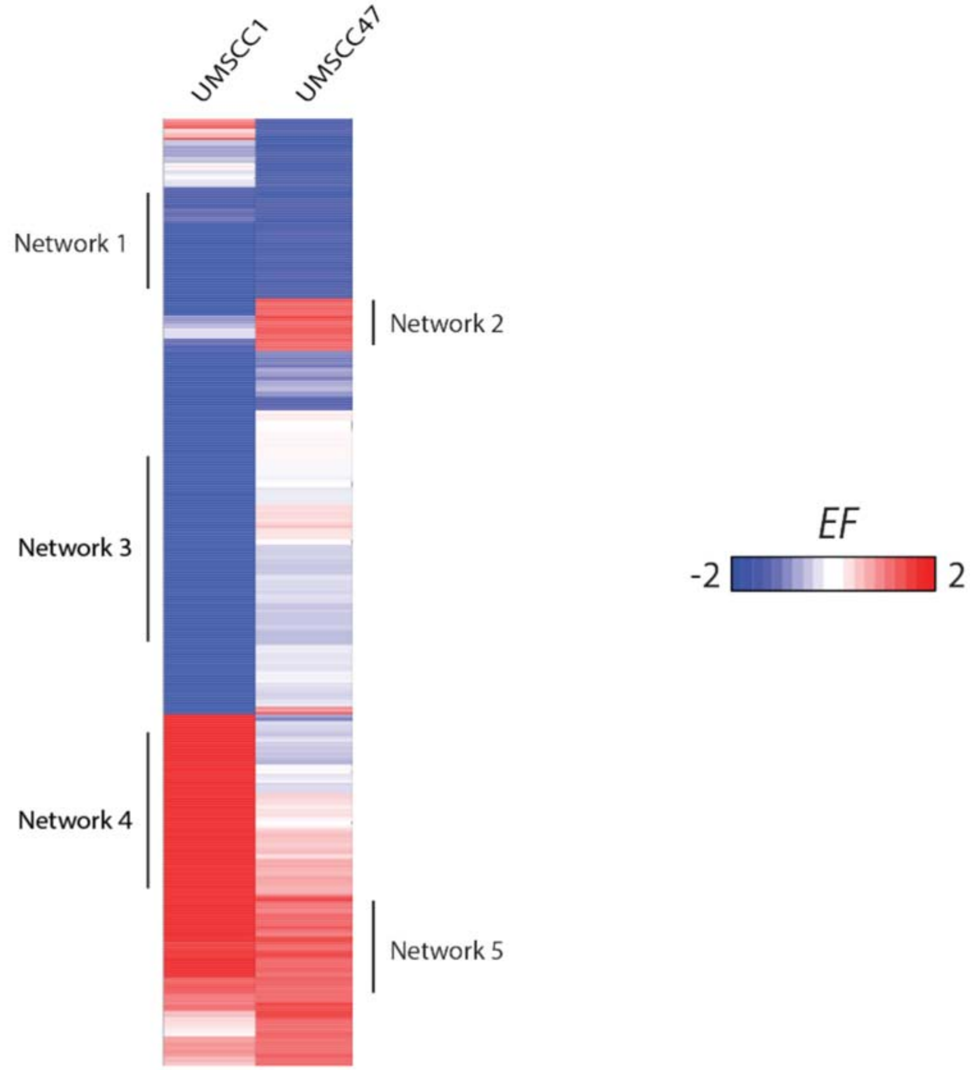

Supplementary Figure S1: Network analysis was performed with Netwalker software on UMSCC1 and UMSCC47 RNA-Seq data. Labeled Networks 1-5 can be seen in expanded views in Supplementary figures 2-6.

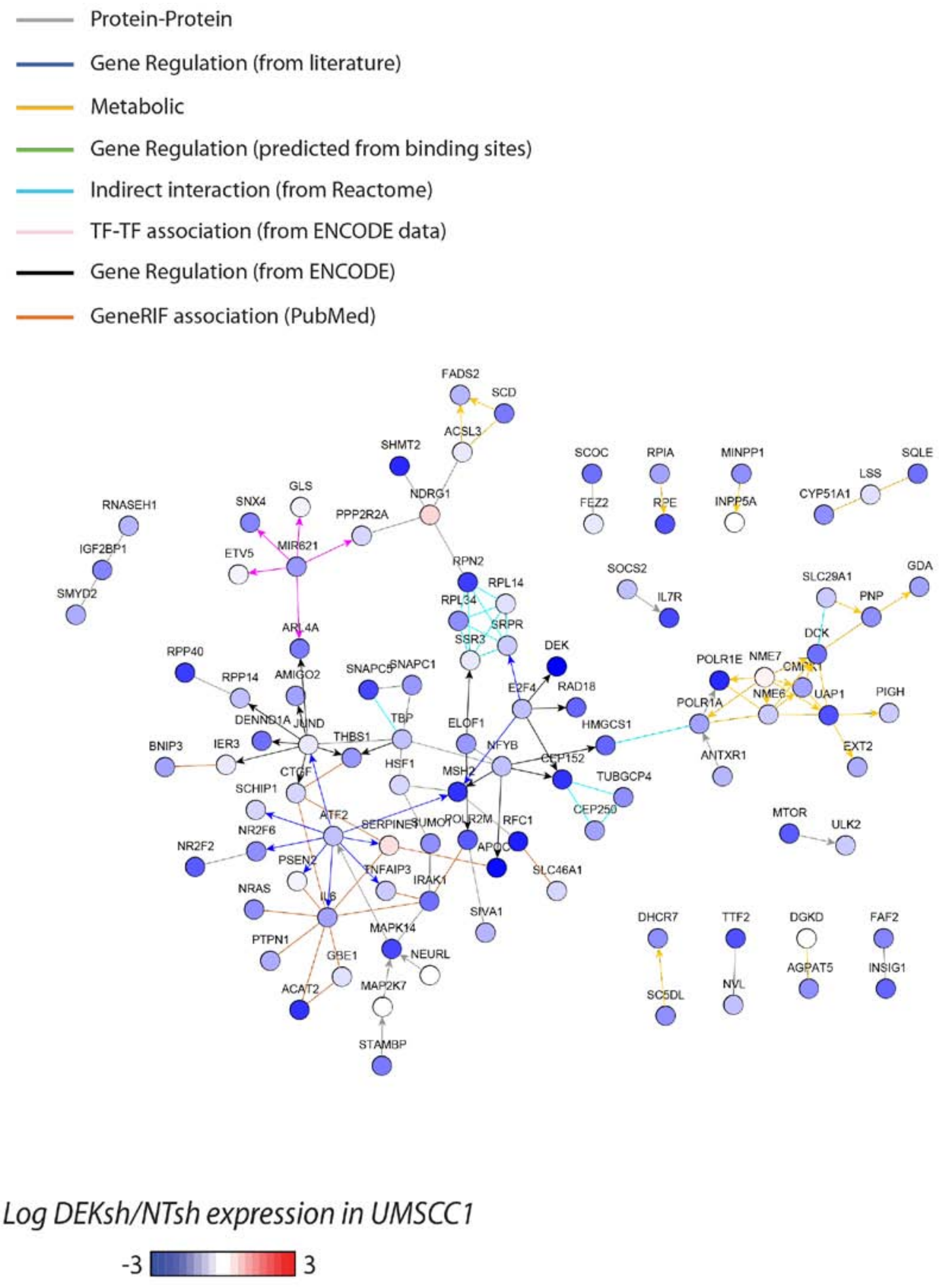

Supplementary Figure S2: Network 1 is represented, with genes down-regulated in UMSCC1, with log fold change represented. This is the expanded view of the network map highlighted in Figure 1.

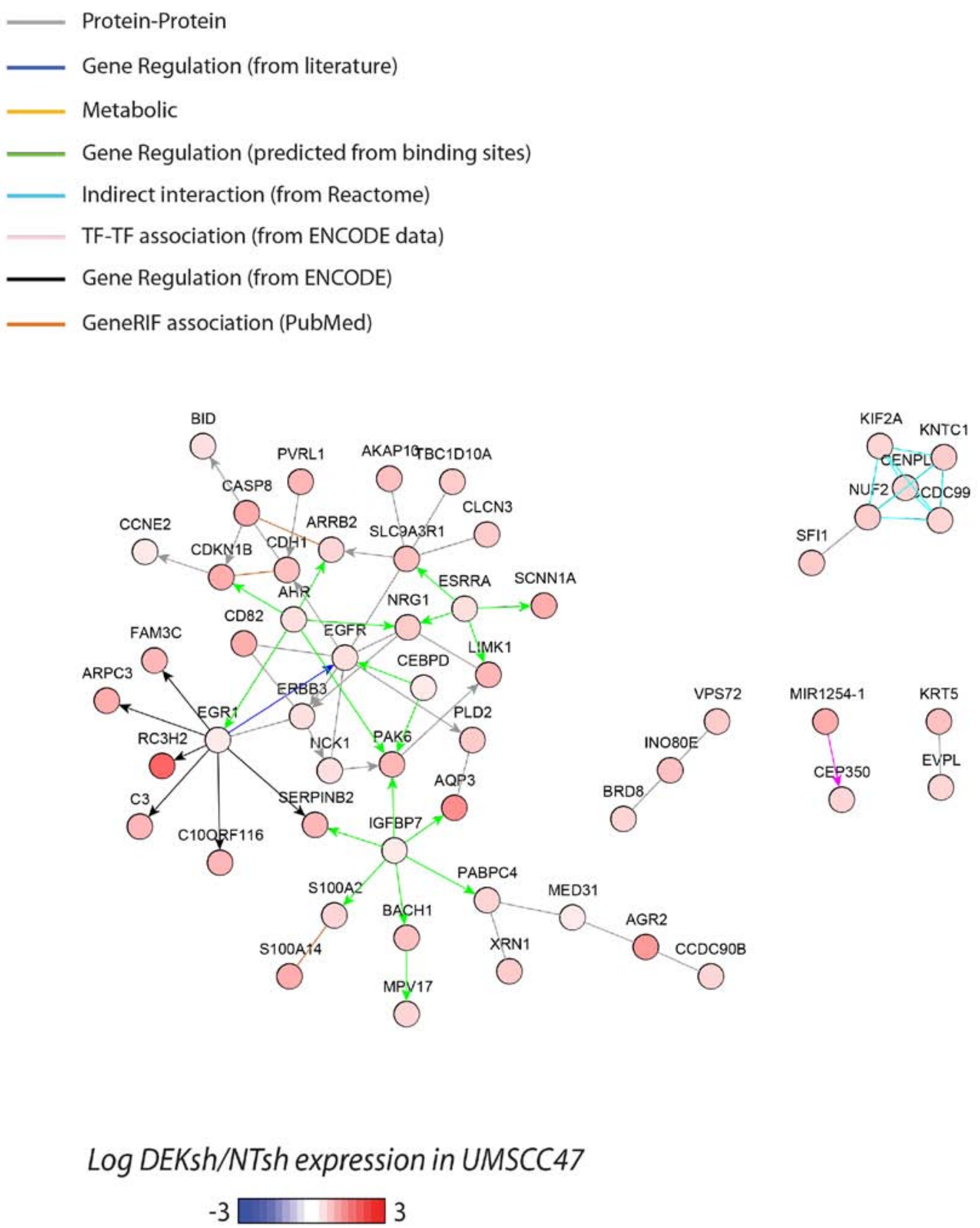

Supplementary Figure S3: Network 2 is represented, with genes up-regulated in UMSCC47, with log fold change represented.

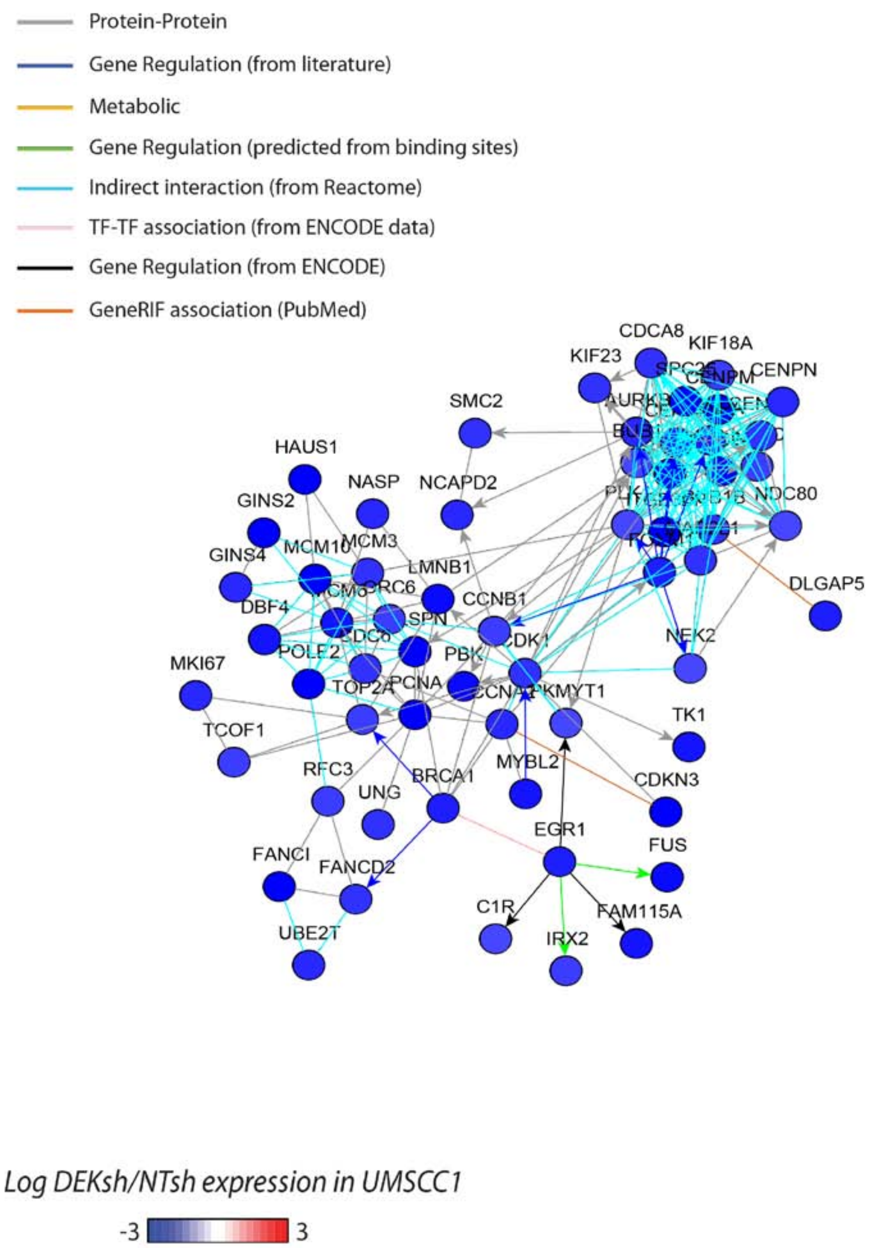

Supplementary Figure S4: Network 3 is represented, with genes down-regulated in UMSCC1, with log fold change represented.

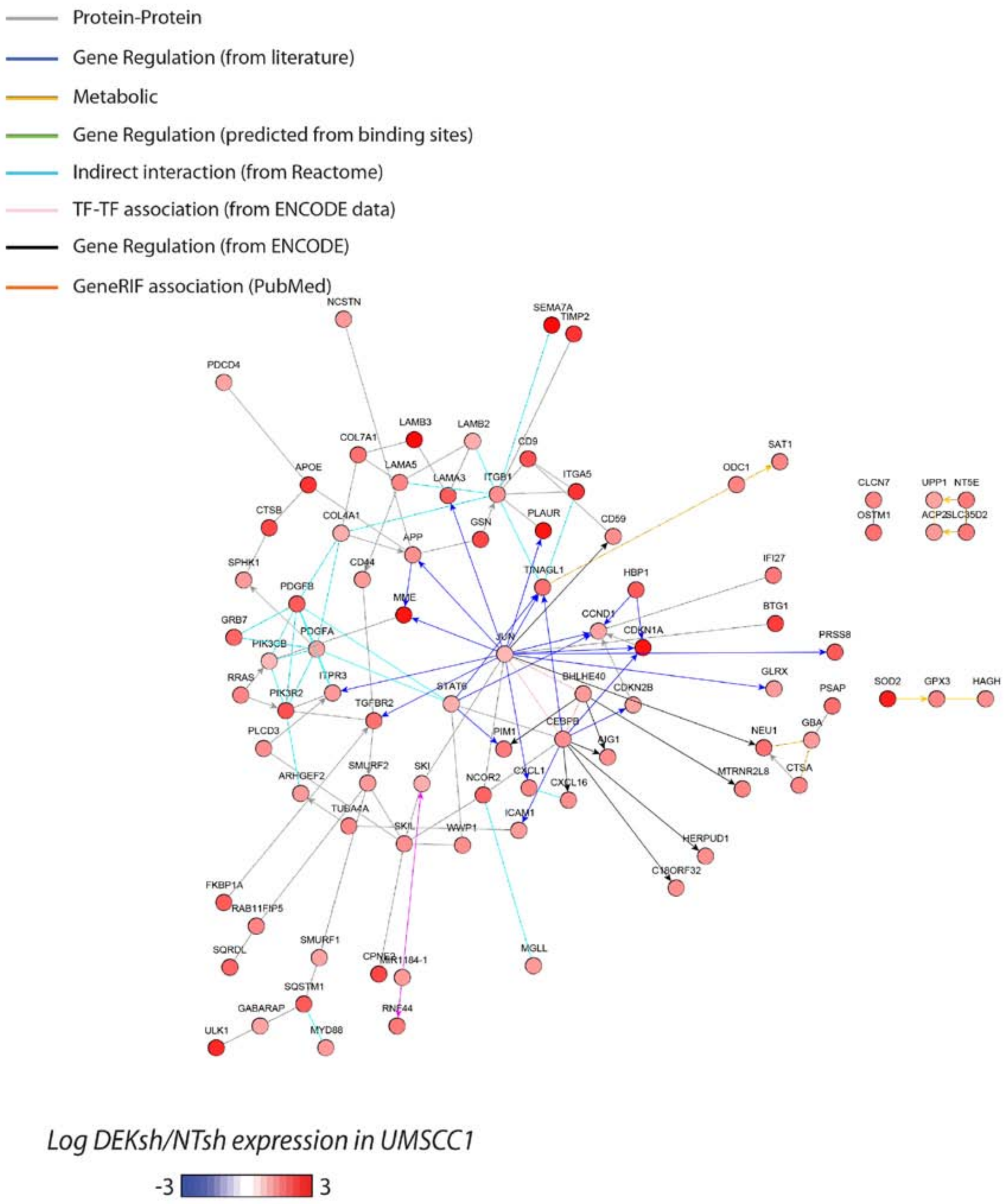

Supplementary Figure S5: Network 4 is represented, with genes up-regulated in UMSCC1, with log fold change represented.

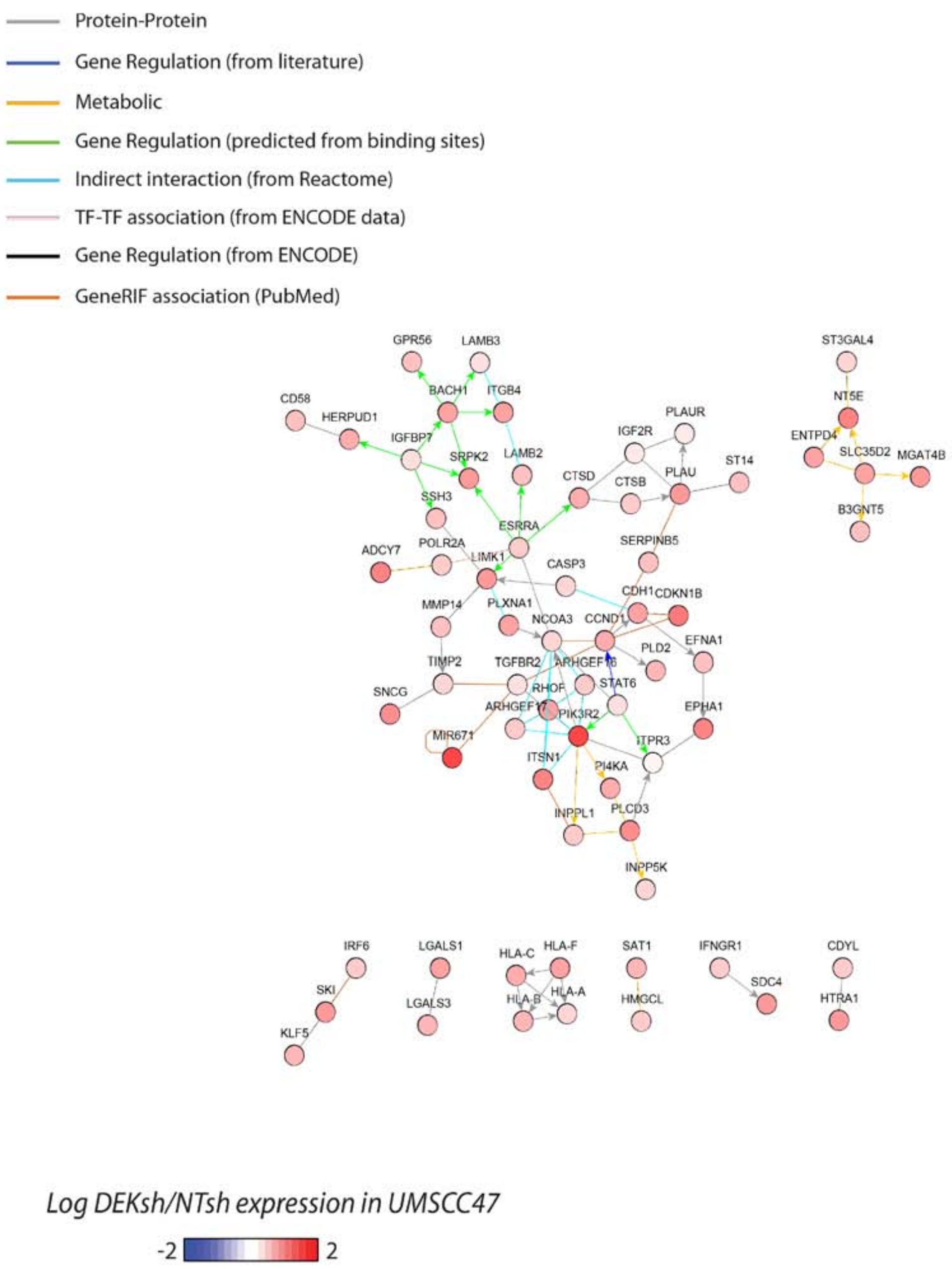

Supplementary Figure S6: Network 5 is represented, with genes up-regulated in UMSCC47, with log fold change represented.

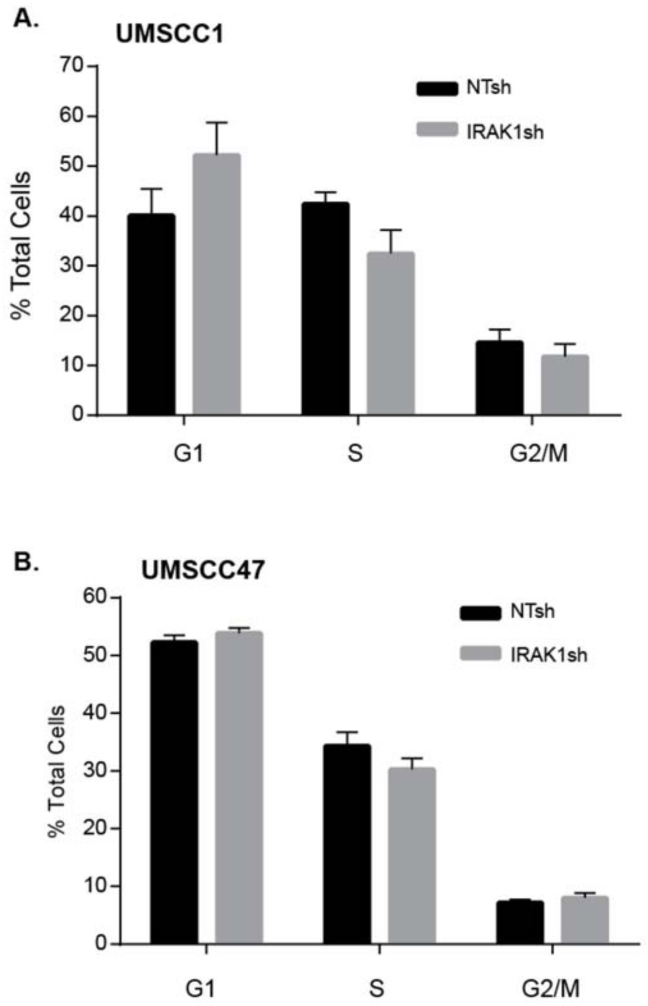

Supplementary Figure S7: UMSCC1 and UMSCC47 cells with IRAK1 knockdown reveals no alterations in cell cycle profiles. BrdU incorporation was measured by flow cytometry to determine G1, S, and G2/M populations. Experiments were performed in triplicate.

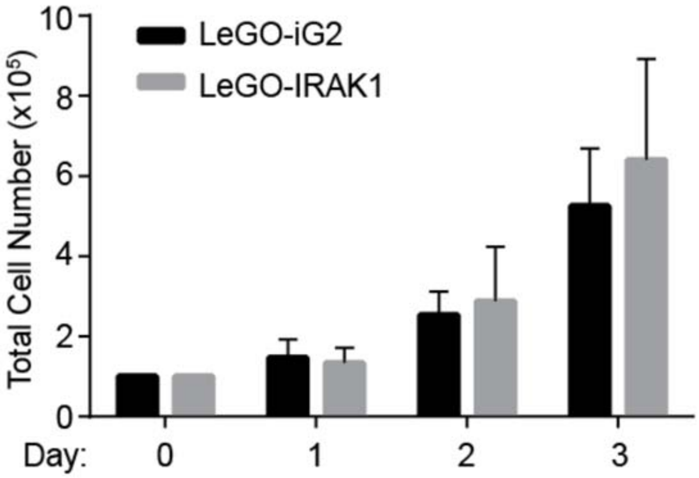

Supplementary Figure S8: UMSCC1 IRAK1 overexpression cells (IRAK1) do not exhibit cell growth difference over controls (iG2). Cells were plated at equal densities and counted over three days. Experiments were performed in duplicate.

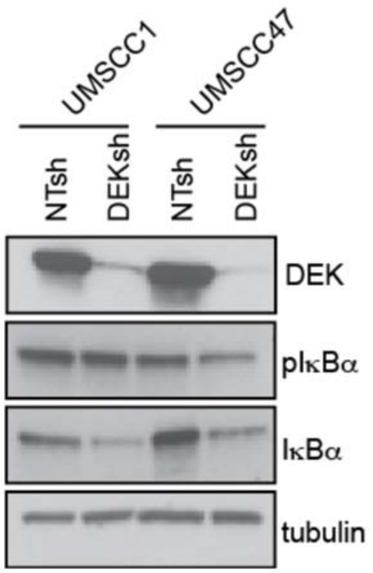

Supplementary Figure S9: UMSCC1 and UMSCC47 control (NTsh) and DEK-deficient (DEKsh) lysates were analyzed by western blot for phospho and total IkBα. Tubulin was used as a loading control.
